# Supplementary material for: Content-rich biological network constructed by mining PubMed abstracts
Source: BMC Bioinformatics. 2004 Oct 8;5:147. doi: 10.1186/1471-2105-5-147 (PMC528731; doi:10.1186/1471-2105-5-147)
Supplement: Additional File 2 — The original results of the above study (non-essential files are deleted to keep the file size under the limit set by BMC bioinformatics). [file 1471-2105-5-147-S2.bz2 › chilibotAdditionalFile2/dip05/396ID10775655E9950/html/EGFR_TGFA.html]

 


 **EGFR** and **TGFA** 
  
Found 1071 abstracts in PubMed, retrieved 05.  
 

 What does Google say? 
 PDF only 
| .edu only 

---

**Interactive relationship** (e.g. stimulation, inhibition, etc)

**Inhibitory relationship**- Our results demonstrate that E4T and  **TGFa**  provoke decreased Cbl recruitment,  **EGFR**  ubiquitination and  **EGFR**  degradation compared to EGF.  Ref: 12829707 J Biol Chem< MedlineTA>J Biol Chem,
- We conclude that the enhanced  **EGFR**  recycling that has been observed in HER 14 cells following  **TGFa**  or E4T stimulation correlates with decreased  **EGFR**  ubiquitination and  **EGFR**  degradation,.  Ref: 12829707 J Biol Chem< MedlineTA>J Biol Chem,
- Co treating PC 3 cells with quercetin significantly attenuated EGF and TGF alpha  [ **TGFA** ]  induced growth and phosphorylation of ErbB  [ **EGFR** ]  2, ErbB  [ **EGFR** ]  3, c Raf, MAPK kinase 1 2 MEK1 2, MAPK, Elk 1 and Akt 1.  Ref: 12888923 Int J Oncol, 2003
**Neutral relationship**- Our simulations predict that when  **EGFR**  is activated with TGF alpha  [ **TGFA** ] , receptor activation is biased toward the cell surface whereas EGF produces a signaling bias toward the endosomal compartment.  Ref: 12885624 Biophys J, 2003

**Non-interactive relationship** (e.g. studied together, co-existance, homology, etc.)

- Similarly,  **EGFR**  TK inhibition by ZD1839 reversed the invasive phenotype promoted by the constitutively activated form of the  **EGFR**  EGFRvIII and the  **EGFR**  agonists transforming growth factor alpha  [ **TGFA** ]  TGFalpha, amphiregulin and EGF.  Ref: 12881705 Oncogene, 2003
- Immunohistochemical procedures were performed to detect the following protein antigens CD99, interleukin IL 1alpha, IL 6, transforming growth factor TGF alpha  [ **TGFA** ] , conventional c protein kinase C cPKC alpha, cPKC betaII, phosphorylated p PKC alpha betaII, c kit CD117, platelet derived growth factor receptor PDGFR alpha, PDGFR beta, epidermal growth factor receptor  [ **EGFR** ]   **EGFR** , human epidermal growth factor receptor  [ **EGFR** ]  HER 2 neu, cathepsin D, angiotensin converting enzyme ACE, angiotensin II type 1 AT1 receptor, p21ras, the alpha subunit of farnesyl and geranylgeranyl transferase FTalpha GGTalpha, phospho p c Jun N terminal kinase p JNK, p p38 mitogen activated protein kinase MAPK, cyclin D1, c Jun, Ki 67, bcl 2, TGF beta1 latency associated peptide LAP, TGF betaRII, and cyclooxygenase COX 2.  Ref: 12817616 Ann Clin Lab Sci< MedlineTA>Ann Clin Lab Sci, 2003
- Inhibition of ErbB  [ **EGFR** ]  2 and ErbB  [ **EGFR** ]  3 expression by quercetin prevents transforming growth factor alpha  [ **TGFA** ]  TGF alpha  [ **TGFA** ]  and epidermal growth factor EGF induced human PC 3 prostate cancer cell proliferation.  Ref: 12888923 Int J Oncol, 2003
- Here we evaluated  **EGFR**  activation, Cbl recruitment,  **EGFR**  ubiquitination and degradation in response to EGF,  **TGFa**  and E4T.  Ref: 12829707 J Biol Chem< MedlineTA>J Biol Chem, 2003
- To determine the utility of this approach, we simulated the differential activation of the  **EGFR**  by two of its ligands, epidermal growth factor EGF and transforming growth factor alpha  [ **TGFA** ]  TGF alpha  [ **TGFA** ] .  Ref: 12885624 Biophys J, 2003
